# CDK 4/6 Inhibitors as Single Agent in Advanced Solid Tumors

**DOI:** 10.3389/fonc.2018.00608

**Published:** 2018-12-12

**Authors:** Francesco Schettini, Irene De Santo, Carmen G. Rea, Pietro De Placido, Luigi Formisano, Mario Giuliano, Grazia Arpino, Michelino De Laurentiis, Fabio Puglisi, Sabino De Placido, Lucia Del Mastro

**Affiliations:** ^1^University of Naples Federico II, Naples, Italy; ^2^Baylor College of Medicine, Houston, TX, United States; ^3^Istituto Nazionale Tumori Fondazione G. Pascale, Naples, Italy; ^4^Department of Medicine, University of Udine, Udine, Italy; ^5^IRCCS Centro di Riferimento Oncologico Aviano, Aviano, Italy; ^6^Policlinico San Martino-IST, Genova, Italy; ^7^University of Genova, Genova, Italy

**Keywords:** solid tumors, cyclin-dependent kinases, palbociclib, ribociclib, abemaciclib, cell cycle

## Abstract

Cyclin-dependent kinases (CDK) 4/6 inhibitors, namely abemaciclib, palbociclib, and ribociclib, interfere with cell cycle progression, induce cell senescence and might promote cancer cell disruption by a cytotoxic T cells-mediated effect. Phase III randomized clinical trials have proven that CDK4/6 inhibitors (CDK4/6i) in combination with several endocrine agents improve treatment efficacy over endocrine agents alone for hormone receptor positive (HR+) HER2 negative (HER2–) metastatic breast cancer (MBC). Based on such results, these combinations have been approved for clinical use. Preclinical studies in cell cultures and mouse models proved that CDK4/6i are active against a broad spectrum of solid tumors other than breast cancer, including liposarcoma, rhabdomyosarcoma, non-small cell lung cancer, glioblastoma multiforme, esophageal cancer, and melanoma. The role of CDK4/6i in monotherapy in several solid tumors is currently under evaluation in phase I, II, and III trials. Nowadays, abemaciclib is the only of the three inhibitors that has received approval as single agent therapy for pretreated HR+ HER2– MBC. Here we review biological, preclinical and clinical data on the role of CDK4/6 inhibitors as single agents in advanced solid tumors.

## Introduction

The key role of cyclin-dependent kinases (CDK) and D-type Cyclins (CCND) in cell cycle progression from G1 to S phase was discovered more than 20 years ago (1). Since then, it has been demonstrated that several solid tumors present direct modifications of genes codifying for several proteins involved in CCND-CDK activity and regulation (2). As a result, in recent years, small molecule inhibitors which target this mitogenic pathway have been developed. Three of them are currently available for the treatment of metastatic breast cancer (MBC) in combination with aromatase inhibitors or fulvestrant. This review focuses on the role of CCND-CDK in normal cells, on how this pathway is altered in solid tumors and on the activity of CDK4/6 inhibitors (CDK4/6i), as single agents in the treatment of advanced solid tumors in adult patients.

## The Role of CDK in Cell Cycle and Solid Tumors

**Figure 1:**
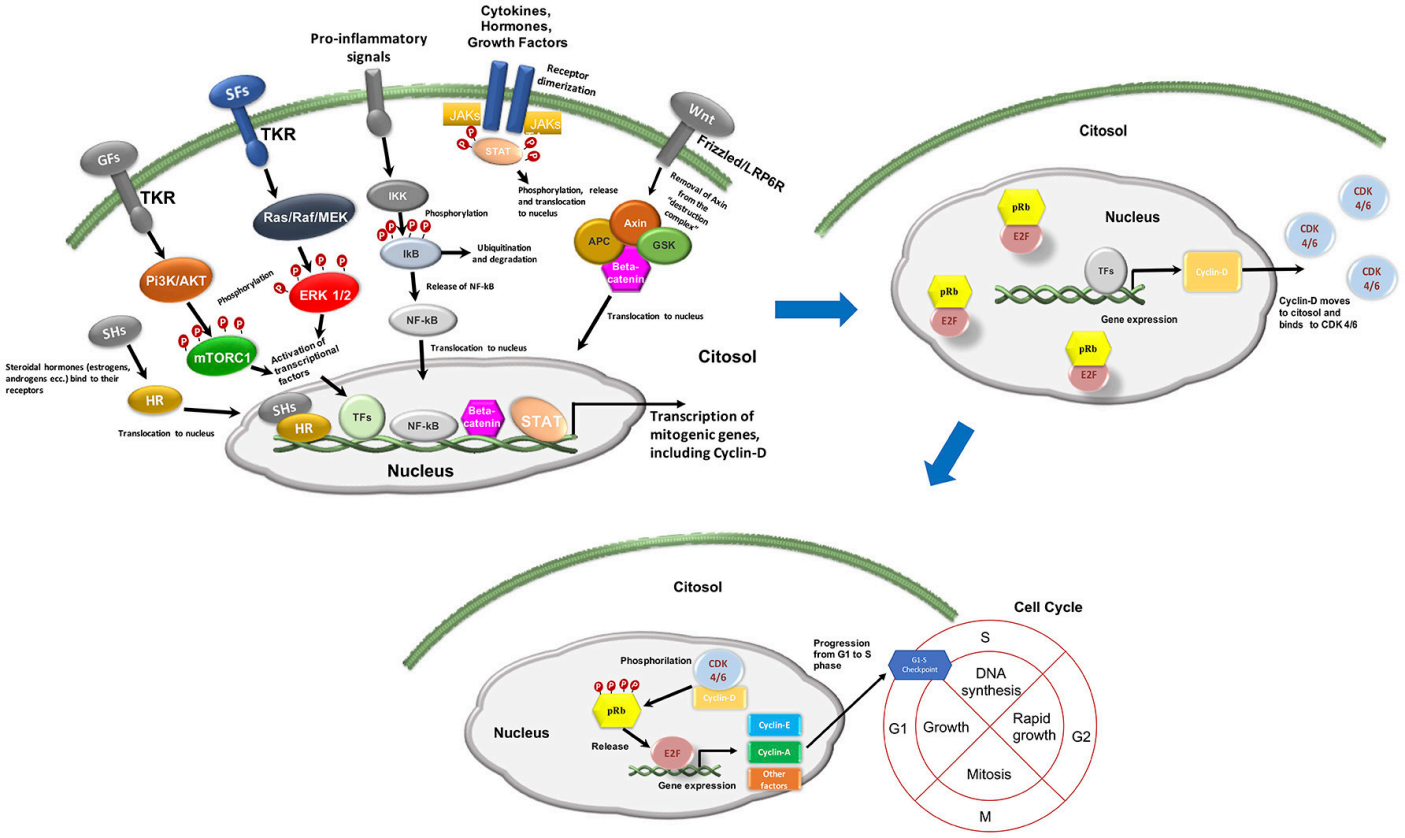
Mitogenic signaling and cell cycle progression. GFs, growth factors; TKR, tyrosine kinase receptor; SFs, survival factors; HR, hormone receptor; SHs, steroidal hormones (i.e., estrogens, androgens); TFs, transcriptional factors.

CCND interact with several CDK, including CDK 4/6, forming functional complexes that phosphorylate and inactivate retinoblastoma protein (pRb) (1). This protein operates a negative control on E2F transcription factors, resulting in an inhibition of cell cycle progression. Indeed, E2F modulates the expression of a broad variety of genes implied in cell cycle S1 phase and mitosis. On the opposite, functional CCND-CDK4/6 complexes allow E2F to be released from pRb control and promote the transition from the G1 to the S phase of the cell cycle (Figure 1) (1). Cyclin D is important in growth factor signaling and, more in general, is a common downstream pathway for several mitogenic signaling, including phosphatidylinositol 3-kinase (PI3K)/AKT/mammalian target of rapamycin (mTOR), mitogen-activated protein kinase (MAPK), wnt/beta-catenin, janus kinase (JAK)-signal transducer and activator of transcription (STAT), nuclear factor kappa-light-chain-enhancer of activated B cells (NF-kB), and steroid hormone signaling pathways (e.g., estrogen, progesterone, and androgen) (Figure 1) (2). CDK 4/6 activity is regulated by the INK4 family of proteins. Among them, p16^INK4A^ appears to be the most relevant, in terms of tumor suppression activity. Several other factors, including p21^CIP1^ and p27^KIP1^ modulate CCND-CDK4/6 complexes' activity in a context-dependent manner (2). Finally, the SMARCB1/INI1/SNF5 tumor suppressor gene directly represses the transcription of the Cyclin-D coding gene CCND1 and increases the expression of CCND-CDK4/6 negative regulators p16^INK4A^ and p21^CIP1^ (2).

In solid tumors, an hyperactivation of the CCND-CDK4/6 activity can occur through: (1) increased activity of upstream mitogenic signaling pathways; (2) aberrant activity of the components of the pathway or their regulators. This latter may depend on various molecular mechanisms, i.e., point mutations, translocations or amplification of CDK4/6, amplification of D-type cyclins, deletions that cause the loss of p16^INK4A^ or pRb expression, epigenetic modifications and downregulation of microRNAs (miRNAs) that target CDK4/6. Alterations of the expression of CCND-CDK4/6-INK4-Rb pathway components or of their direct regulators result in cell cycle progression and cell proliferation and represent a key mechanism of tumorigenesis (2). The solid tumors for which the CCND-CDK4/6-INK4-Rb pathway is more frequently deregulated through direct genetic, epigenetic or transcriptional modifications are breast, head and neck, lung, pancreatic, ovarian and bladder cancer, melanoma, endometrial carcinoma, liposarcoma, neuroblastoma, and malignant rabdoid tumors (3–25). Because of their central role in tumorigenesis and progression, CDK4 and 6 might represent a valid therapeutic target for cancer treatment in a broad spectrum of solid tumors.

## CDK 4/6 Inhibitors: An Overview

### Mechanism of Action and Toxicities

**Figure 2:**
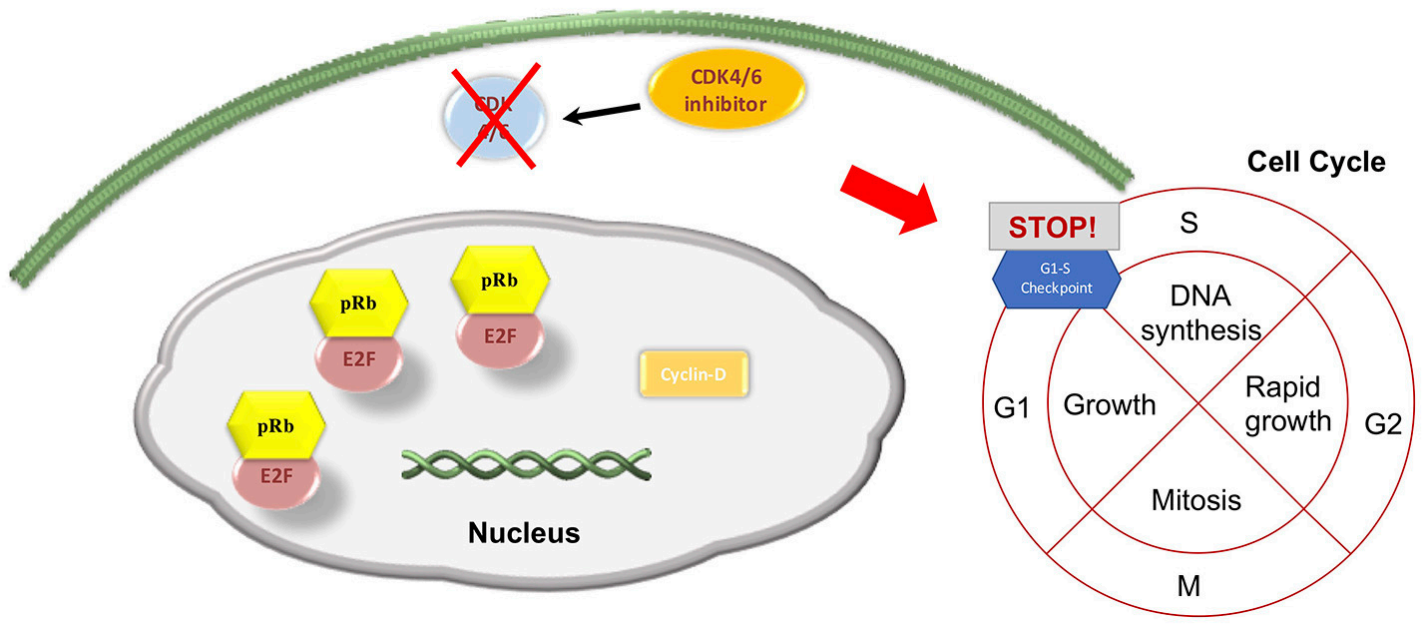
CDK4/6 inhibitors' main effect on cell cycle progression.

After the discovery of CDK 4/6 role in tumorigenesis, several CDK inhibitors have been developed for clinical use. The most recent are selective for CDK4 and CDK6, preventing inhibition of other CDKs activity (1). Three CDK4/6i are currently approved in clinical practice, namely: palbociclib, ribociclib, and abemaciclib. Their mechanism of action is based on the binding to CDK 4 and 6 ATP pocket, which leads to a substantial inactivation of CCND-CDK4/6 complexes, with a subsequent increase in the activity of pRb. The logic consequence is a G1 phase arrest (Figure 2). The interference with cell cycle progression results in an increased apoptosis phenomena in tumor cells (1, 2).

Palbociclib and ribociclib are similar in chemical structure, while abemaciclib differs and has a higher CDK4/6 binding power than the other two CDK4/6i. More specifically, abemaciclib shows higher selectivity for the complex CDK4/cyclin D1 compared to the other two compounds, and is 14 times more potent against CDK4 than CDK6 (2, 26). Cell cycle arrest and subsequent apoptosis are sought to be the most relevant mechanism of action of CDK4/6i. However, a very recent study based on mouse models of breast cancer and other solid tumors and on a confirmatory trascriptomic analysis of serial biopsies from a clinical trial involving CDK4/6i in breast cancer, showed that CDK4/6 inhibition might also induce a broad spectrum of immunologic events. More precisely, they seem to increase the antigen presenting capability of tumor cells, while concurrently reducing the immunosuppressive population of T regulator lymphocytes. This could in turn enhance the activation of cytotoxic T cells, which ultimately kill tumor cells (27). However, immunologic effects of CDK4/6i are still object of debate and need further validation/confirmation. Despite a very similar mechanism of action, dose limiting toxicities (DLTs) observed in phase I trials differed, with neutropenia being the DLT for palbociclib, diarrhea and fatigue for abemaciclib, and neutropenia, mucositis, asymptomatic thrombocytopenia, pulmonary embolism, increased creatinine, hyponatriemia, and QTcF prolongation for ribociclib (2, 28). Some of the latter toxicities (such as creatinine increase or thromboembolic events) were also reported for abemaciclib however they did not represent formal DLTs in phase I trials. The most common CDK4/6i toxicities of any grade observed in pivotal phase III trials were neutropenia, leukopenia, fatigue and nausea for palbociclib (29, 30), neutropenia, nausea, infections, fatigue and diarrhea for ribociclib (31, 32), creatinine increase, diarrhea, fatigue, and neutropenia for abemaciclib (33, 34). The pathophysiology of such toxicities has mostly to be linked to CDK4/6i mechanism of action. Additionally, abemaciclib-induced creatinine increase, might be due to its competitive inhibition of efflux transporters of creatinine (26). A comparison between main pharmacokinetic and pharmacodynamic properties among the three molecules is reported in Table 1. All of the three molecules are orally administered and are metabolized by the liver. Palbociclib and ribociclib, due to longer half-life than abemaciclib, can be administered once daily, while abemaciclib needs twice daily administration.

**Table 1:** CDK 4/6 inhibitors' pharmacological characteristics.

**Drug properties**	**CDK 4/6 inhibitors**		
	**Palbociclib**	**Ribociclib**	**Abemaciclib**
Bioavailability (35)	46%	Unknown	45%
Protein binding (35)	85%	~70%	96.3%
Metabolism (35)	Liver	Liver	Liver
Elimination half-life (35)	29 (±5) h	32.0 (29.7–54.7) h	18.3 h
Excretion (35)	74% feces, 18% urine	69% feces, 23% urine	81% feces, 3% urine
**IC50 (nM)** (2)			
➢ CDK4-cyclin D1	11	10	2
➢ CDK6-cyclin D1-2-3	15	39	10
➢ CDK1-cyclin B	>10,000	113,000	1,627
➢ CDK2-cyclin A-E	>10,000	76,000	504
➢ CDK9-cyclin T	NR	NR	57
MTDs (2)	125 mg	900 mg	200 mg every 12 h
DLTs (2)	Neutropenia	Neutropenia, Mucositis, Asymptomatic thrombocytopenia, Pulmonary embolism, Increased creatinine, Hyponatriemia, QTcF prolongation (>500 ms)	Fatigue
Recommended dose (35)	125 mg/die on a 21-on-28-days schedule	600 mg/die on a 21-on-28-days schedule	200 mg twice daily
Administration (35)	Oral	Oral	Oral

### Current Indications

The three inhibitors are currently available for the treatment of hormone receptor positive (HR+) Human Epidermal Growth Factor Receptor 2 negative (HER2–) MBC in combination with an aromatase inhibitor (AI) as first-line endocrine therapy or in combination with fulvestrant in pretreated patients. All of these combinations substantially doubled the comparator in terms of median progression-free survival (PFS) (29–34). Moreover, ribociclib was also studied in combination with tamoxifen or AIs and a GnRH analog (GnRHa) in pre-/perimenopausal setting in the context of the MONALEESA 7 phase III trial (36), which enrolled HR+ HER2– MBC in first line setting and results were in line with those published in the other CDK4/6i pivotal trials. Results and characteristics of the pivotal trials, namely PALOMA 2 and 3, MONALEESA 2, 3, and 7, and MONARCH 2 and 3 are reported in Table 2.

**Table 2:** Characteristics of pivotal trials concerning CDK4/6 inhibitors approved for clinical practice.

**Characteristics**	**Pivotal trials**						
	**Paloma 2 (29)**	**Paloma 3 (30)**	**Monaleesa 2 (31)**	**Monaleesa 7 (36)**	**Monaleesa 3 (32)**	**Monarch-3 (33)**	**Monarch-2 (34)**
Combination	Palbociclib + letrozole vs. letrozole	Palbociclib + fulvestrant vs. fulvestrant	Ribociclib + letrozole vs. letrozole	Ribociclib + tamoxifen or AI + GnRHa vs. tamoxifen or AI + GnRHa	Ribociclib + fulvestrant vs. fulvestrant	Abemaciclib + NSAI vs. NSAI	Abemaciclib + fulvestrant vs. fulvestrant
Menopausal status	Post-menopausal (iatrogenic or physiologic)	Post-menopausal (iatrogenic or physiologic)	Post-menopausal	Pre- and perimenopausal	Post-menopausal	Post-menopausal (iatrogenic or physiologic)	Post-menopausal (iatrogenic or physiologic)
Setting	1st line HR+ HER2– MBC	≥1st line HR+ HER2– MBC	1st line HR+ HER2– MBC	1st line HR+ HER2– MBC	≥1st line HR+ HER2– MBC	1st line HR+ HER2– MBC	1st line HR+ HER2– MBC
Median PFS (months)	24.8 vs. 14.5	9.5 vs. 4.6	NR vs. 14.7	23.8 vs. 13.0	20.5 vs. 12.8	NR vs. 14.7	16.4 vs. 9.3
PFS HR (95% Cis); *p*-value^*^	0.58 (0.46–0.72); *p* < 0.001	0.46 (0.36–0.59); *p* < 0.0001	0.56 (0.43–0.72); *p* = 3.29 × 10^−6^	0.553 (0.441–0.694); *p* < 0.0001	0.59 (0.48–0.73); *p* = 4.10 × 10^−7^	0.543 (0.409–0.723); *p* = 0.000021	0.553 (0.449–0.681); *p* = 0.000021
ORR	42.1 vs. 34.7%	25 vs. 11%	40.7 vs. 27.5%	51 vs. 36%	41 vs. 9%	59.2 vs. 43.8%	48.1 vs. 21.3%
Trial phase	III	III	III	III	III	III	III
FDA/EMA status	A/A	A/A	A/A	A/NA	A/NA	A/A	A/A

* *OS data not mature, yet, except for palbociclib + fulvestrant vs. fulvestrant [HR 0.81 (0.64–1.03); p = 0.043] (37)*.

*NSAI, non-steroidal aromatase inhibitor; AI, aromatase inhibitor; GnRHa, gonadotropin releasing hormone analog; HR+, ER and/or PgR positive; HER2–, human epidermal growth factor receptor 2 negative; A, approved; NA, not yet approved*.

## Single Agents CDK4/6i: Current Evidence

As previously reported, the CCND-CDK4/6-INK4-Rb pathway is frequently deregulated through direct genetic, epigenetic or transcriptional modifications in a broad variety of neoplasms (3–25). Indeed, apart from their use in combination with ET for the treatment of HR+ HER2– MBC, CDK4/6i are also under study as single agent in breast cancer (BC) and other solid tumors. The following paragraphs will resume the current preclinical and clinical evidence supporting this experimental treatment strategy.

### Preclinical Evidence

Single agent CDK4/6i have shown consistent activity in preclinical models (38–56). In brief, the most relevant results were observed in *in vivo* and/or *in vitro* models of colon cancer (palbociclib, abemaciclib), glioblastoma (palbociclib, abemaciclib), breast cancer (palbociclib, ribociclib, abemaciclib), prostate carcinoma (palbociclib), sarcomas (palbociclib and ribociclib), pancreatic ductal adenocarcinoma (palbociclib), melanoma (palbociclib, ribociclib, abemaciclib), non-small cell lung cancer (palbociclib, abemaciclib), and esophageal adenocarcinoma (abemaciclib).

#### Palbociclib

A study demonstrated a potent antitumor activity in different mice models, bearing colon cancer, glioblastoma, breast, and prostate carcinoma xenografts. Palbociclib, given as continuous treatment, was able to arrest growth and induce regression of tumor xenografts. A modest activity was also observed in non-small cell lung cancer (NSCLC) models (38). Palbociclib was also able to arrest the growth of estrogen receptor-positive (ER+) BC cell lines (39). A potent antitumor activity was also demonstrated in an *ex vivo* model of human breast tumors (40). Palbociclib activity was demonstrated on cell lines and intracranial xenografts of glioblastoma multiforme (GBM) (41). In the latter case, the proneural subtype appeared to be the most sensitive to palbociclib activity (42). In ovarian cancer cell lines, Palbociclib induces G0/G1 cell cycle arrest by reducing pRb phosphorylation (43). Palbociclib is also effective in arresting cell cycle progression and blocking proliferation in synovial sarcomas cell lines (44). Another study demonstrated that palbociclib may inhibit cellular growth and induce senescence in liposarcoma cell lines and mice xenografts (45) and in sarcoma cell lines (46). An antiproliferative effect was observed also in rhabdomyosarcoma-derived cell cultures (47). Palbociclib was also studied in immunocompromised mice with subcutaneous and intrasplenic injections of pancreatic ductal adenocarcinoma (PDA) cell lines derived from patients' specimens. The CDK 4/6i significantly disrupted extracellular matrix organization and increased quiescence and apoptosis, decreased invasion, metastatic spread and tumor progression (48).

#### Ribociclib

Ribociclib as single agent is effective in inhibiting cell growth in liposarcoma cell lines. Moreover, the administration to mice bearing human liposarcoma xenografts resulted in tumor growth inhibition and/or tumor regression. A similar effect was noted in preclinical models of breast cancers with intact estrogen receptor and/or activating aberrations of PIK3CA/HER2 (49). In preclinical models, ribociclib also showed some activity in melanomas with activating mutations of BRAF or NRAS (50).

#### Abemaciclib

Abemaciclib is effective in inducing cell cycle arrest and tumor growth inhibition in colon cancer and breast cancer cell lines and in mice bearing human melanoma and colon cancer xenografts (51, 52). Abemaciclib, similarly to temozolamide, increased survival in a rat xenograft model of glioblastoma (53), thus suggesting a significant capability to cross the blood-brain barrier (BBB). It was also effective on NSCLC tumor xenografts (54). Abemaciclib was also able to inhibit growth of melanoma tumor xenografts and delay tumor recurrence in combination with vemurafenib. Furthermore, abemaciclib yielded tumor growth regression in a vemurafenib-resistant model, and induced apoptotic cell death in a concentration-dependent manner, suggesting that this drug might be a viable therapeutic option to overcome MAPK-mediated resistance to B-RAF inhibitors in B-RAF V600E melanoma (55). Abemaciclib was also evaluated in preclinical models of esophageal adenocarcinoma (EAC): in tumor cell lines it appeared to increased apoptosis and decrease proliferation while in mice models, it was able to decrease of more than 20% tumor volume (56).

### Clinical Evidence

The preclinical data reviewed above offered a solid rationale to test single agent CDK4/6i in clinical trials.

#### Palbociclib: Completed Trials

Palbociclib was tested in a cohort of 41 patients affected by several solid tumors in the context of a phase I dose escalating study. Tumors had been screened for the presence of pRb. In this trial the maximum tolerated dose (MTD) and recommended phase II dose (RP2D) of single-agent palbociclib was 125 mg/day on a 21-of-28 days schedule. The most frequent G3/4 toxicities were neutropenia, leucopenia and anemia with the first present in 20% of cases, the second in 10% and the latter in 7% of cases. Albeit being a phase I trial, clinical activity was also reported. Among 37 evaluable patients, 27% achieved stable disease (SD) for at least 4 cycles and 16% for at least 10 cycles (57).

Several phase II studies tested palbociclib monotherapy in a broad variety of solid tumors, namely well-differentiated or dedifferentiated liposarcoma (WD/DDLS) (58, 59), NSCLC (60), gastric and esophageal cancer (61), urothelial carcinoma (62), epithelial ovarian cancer (63), HR+ and triple negative (TN) BC (64, 65). The best results were observed in WD/DDLS, ovarian and BC, counterbalanced by overall disappointing results in the other neoplasms. The most frequent grade (G)3/4 adverse reactions (ADRs) were hematologic.

More in details, a phase II study explored the activity and safety of palbociclib on a 200 mg/day on a 14-of-21-days schedule in patients with advanced CDK4-amplified WD/DDLS. The trial enrolled 30 patients. The estimated 12-weeks PFS rate was 66%, far exceeding the expected rate of 40% for an active agent. There was only one partial response (PR) and 19 SD at 12 weeks. Median PFS (mPFS) was 17.9 weeks. The most frequent G3/4 ADRs were neutropenia (50%), leukopenia (47%), thrombocytopenia (30%), lymphopenia (27%), and anemia (17%) (58). In a subsequent study, patients affected by advanced WD/DDLS were treated with standard palbociclib 125 mg for 21 days in 28 days-schedule. The trial results showed a successful PFS at 12 weeks of 57.2% [95% Confidence Interval (CI): 42.4–68.8%]. The median PFS was 17.9 weeks (95% CI: 11.9–24.0 weeks). One complete response (CR) was observed. G3/4 ADRs were primarily hematologic and included neutropenia (33%), without neutropenic fever (59). A clinical trial in previously-treated patients with recurrent or metastatic NSCLC was prematurely halted due to lack of objective tumor responses. Half of evaluable patients achieved SD. The mPFS was 12.5 weeks. One patient experienced G3 transaminitis and unexpected G4 rhabdomyolysis, supposedly due to concomitant use of high-dose simvastatin. Some patients developed G3 or 4 neutropenia, and G3 thrombocytopenia (60). Single agent palbociclib was also not effective in advanced gastric and esophageal tumors, even if the patients had been selected for Rb expression and despite 19/38 tumors showed amplification of CCND1. The median duration of treatment was of 1.7 months, with a maximum of 5.5 months. No objective responses were observed (61). Similarly, palbociclib was not effective in a phase II trial conducted in patients affected by metastatic urothelial carcinoma with both p16 loss and pRb expression (62). A single arm phase II trial in patients with heavily pretreated epithelial ovarian cancer showed a discreet activity and efficacy for palbociclib as single agent. Thirty percent of patients were progression-free at 6 months, with a median PFS of 3.7 months (95% CI: 1.2–6.2). A 4% of PR and a 65% of SD were observed. The toxicity was minimal. Predictive biomarker analyses are ongoing (63). A phase II study of palbociclib as single agent was conducted in patients with metastatic pRb positive BC. The clinical benefit rate (CBR) at 6 months, composed of all complete responses (CR), PR and SD observed as best responses, was 21%, the median PFS were of 4.1 months (95% CI 2.3–7.7) for patients with ER+ HER2– BC, 18.8 months (95% CI: 5.1—NE) for ER+ HER2+ patients and 1.8 months (95% CI: 0.9—NE) for patients with triple negative (TN) tumors, respectively. Neutropenia (50%) and thrombocytopenia (21%) were the most frequent G3/4 toxicities (64). The TREND study, an Italian multicentre open-label phase II trial, compared single agent palbociclib with palbociclib combined with the same ET received prior to disease progression in post-menopausal women with HR+ HER2– MBC. The trial enrolled 115 patients, the primary endpoint was CBR. In both arms, 67% of pts had the study treatment as second line, 33% as third line, and about 1/3 of pts also had received 1 prior chemotherapy for MBC. The CBR was similar in both arms, 54% (95% CI: 42–67%) observed in the combination one, and 60% (95% CI: 48–73%) with palbociclib alone. The Overall Response Rates (ORR), composed of all CR and PR observed as best responses, were 11% (95% CI: 3–19%) and 7% (95% CI: 0.4–13%) with the combination therapy and palbociclib alone, respectively. The trial was not powered to estimate survival endpoints, however exploratory analyses were performed, with no significant differences observed in PFS (*p* = 0.13) and a longer median duration of clinical benefit for the combination than for the single agent [11.5 months, 95% CI: 8.6–17.8 vs. 6 months, 95% CI: 3.9–9.9; Hazard Ratio (HR): 0.31, 95% CI: 0.1–0.7, *p*-value 0.001]. Overall, however, the primary endpoint did not differ significantly between the 2 study arms, thereby lending support to the potential use of palbociclib as single agent in pretreated patients with HR+ HER2– MBC (65).

#### Palbociclib: Ongoing Trials

A number of trials are currently ongoing with single agent palbociclib in several advanced solid tumors.

Results are awaited from the NCT03219554 single arm phase II trial that is evaluating the efficacy of single agent palbociclib in patients with recurrent or metastatic advanced thymic epithelial tumors pretreated with one or more cytotoxic chemotherapy. The primary endpoint is PFS (66). The activity and efficacy of single agent palbociclib will be also evaluated in the Lung-MAP trial, a phase II/III biomarker-driven study for second line therapy of squamous cell lung cancer (SCLC). More specifically, single agent palbociclib will be studied in the context of a sub-study that includes all patients that harbored genetic alterations involving cell-cycle genes. The accrual has been completed and results are awaited (67). A phase II study, the NCT01907607—CYCLIGIST, has also already completed accrual and will evaluate the efficacy of single agent palbociclib in patients with gastrointestinal stromal tumors (GIST) refractory to imatinib and sunitinib. The primary endpoint is the non-progression rate at 4 months (68). Results are also awaited for the NCT01356628. This multicenter single arm phase II trial is exploring the efficacy of single agent palbociclib in advanced hepatocellular carcinoma pretreated with standard therapies. The primary endpoint is the time to disease progression (TTP) (69). Another phase II trial, the NCT02806648—PALBONET, is ongoing to demonstrate the safety and activity of palbociclib in subject affected by pNET with overexpression of CDK4, RB1, and CCND1. Results are awaited (70).

Several trials are currently recruiting participants. The NCT02530320 phase II study is ongoing in patients with oligodendroglioma or recurrent anaplastic oligoastrocytoma with preserved pRb activity. The primary end point is the PFS rate at 6 months (71). Another ongoing single arm phase II study (NCT03242382) will evaluate the efficacy of second-line palbociclib in patients with advanced soft tissue sarcomas with CDK4 overexpression. The primary endpoint is the PFS at 6 months (72). The NCT01037790 phase II clinical trial is studying activity, safety and tolerability of single agent palbociclib in preatreated refractory solid tumors, including metastatic colorectal cancer that harbors the Kras or BRAF mutation, metastatic breast cancer, advanced or metastatic esophageal and/or gastric cancer, cisplatin-refractory, unresectable germ cell tumors and any tumor type if tissue tests positive for CCND1 amplification, CDK4/6 mutation, CCND2 amplification or any other functional alteration at the G1/S checkpoint. Co-primary endpoints are the response rates and the safety and tolerability profile. The trial is currently recruiting participants (73).

Finally, a single arm phase II trial (NCT03454919) in acral melanoma bearing alterations in cell cycle pathways, including CDK4 amplification and/or CCND1 amplification and/or P16 (CDKN2A) loss, is going to start but not yet recruiting patients. The primary end point is PFS (74).

#### Ribociclib: Completed and Ongoing Trials

The initial phase I dose escalation study of single-agent ribociclib enrolled 128 patients with pRb positive advanced solid tumors and lymphomas. The MTD and RP2D were established as 900 and 600 mg/day, respectively, on a 21-of-28-days schedule. The most relevant G3/4 ADRs were neutropenia (27%), leukopenia (17%), fatigue (2%), and nausea (2%). An asymptomatic QTcF prolongation was observable, but mostly with doses ≥600 mg/day (9% of patients at 600 mg/day; 33% at doses >600 mg/day). Response rates were evaluable for 110 patients, though this was a phase I trial. There were 3 PR and 43 SD as best response; eight patients were progression-free for more than 6 months (75). Results are awaited for an ongoing phase I study (NCT02345824) that will assess tumor pharmacokinetics and efficacy of ribociclib in patients with recurrent glioblastoma or anaplastic glioma (76).

Several phase II trials of single agent ribociclib are currently ongoing. More specifically, the NCT02571829 trial is assessing the efficacy and safety of ribociclib in patients with advanced WD/DDLS. Patients' recruitment has been completed (77). Another trial is ongoing in patients with advanced neuroendocrine tumors of foregut origin progressed after prior systemic therapy. The primary endpoint is the objective response rate (78). The NCT02300987 randomized study is ongoing in patients with relapsed, refractory, incurable teratoma with recent progression from at least 1 prior line of chemotherapy and for which no additional standard surgical or medical therapy exists. This trial will compare ribociclib to placebo. The primary endpoint is PFS. Recruitment has been completed and results are awaited (79). Another phase II single arm study (NCT03096912) assessing efficacy and safety of ribociclib in patients with advanced WD/DDLS is currently recruiting patients. The primary endpoint is the response to therapy after 36 months, as evaluated by RECIST and Choi criteria (80).

#### Abemaciclib: Completed Trials

Abemaciclib as single agent was investigated in a multicentre phase I study conducted by Patnaik and colleagues. In this study, the 225 enrolled patients were affected by NSCLC, BC, melanoma, colorectal cancer and GBM. The MTD was 200 mg twice daily and the DLT was G3 fatigue. The most relevant G3 ADRs were diarrhea (5%), nausea (4%), fatigue (7%), vomiting (2%), and neutropenia (7%). Activity data were also reported. Fifteen patients experienced SD for at least 4 cycles, with 3 patients achieving SD for 8, 16, and 26 cycles, respectively. One patient with ovarian cancer had a durable and relevant CA125 response. One patient with KRAS mutant NSCLC had a PR. One patient with NRAS mutant melanoma had a confirmed PR. The ORR was 31% for HR+ BC. Moreover, when also considering patients who achieved SD as a best response, 61% of the overall subjects obtained a clinical response lasting at least 6 months (81, 82). A focus on 49 NSCLC patients was also published. The most relevant G3 ADRs were diarrhea (2%), nausea (4%), fatigue (2%), vomiting (2%), and anemia (2%); there were no G4 events. Activity results were also shown. The disease control rate (DCR = CR + PR + SD) was 51% with 1 confirmed PR. The median duration of SD was 5.6 months and the median PFS was 2.1 months. Twenty patients reached at least 4 cycles and 13 reached at least 6 cycles. Among those 49 patients, 19 were affected by KRAS wildtype tumors, 26 by KRAS mutant tumors and 4 with unknown KRAS status. The DCR was 37% for KRAS wildtype and 54% for KRAS mutant NSCLC, consistently with what observed in xenograft studies. The MTD was 200 mg twice daily (83). A randomized phase III study JUNIPER, has compared abemaciclib plus best supportive care to erlotinib plus best supportive care in patients with metastatic NSCLC with a detectable KRAS mutation who have progressed after platinum-based chemotherapy. The primary endpoint was OS and the study failed to show a significant benefit. Moreover, researchers reported a higher-than-expected OS rate in the control group based on historical data (84, 85).

At present, the most relevant trial involving abemaciclib in monotherapy is the MONARCH-1. Such study was a single arm phase II trial in which the efficacy and safety profile of abemaciclib as a single agent were investigated in HR+ HER2– MBC. The 132 enrolled patients had to be progressed on or after prior ET and must have received at least two prior chemotherapy regimens, at least one but no more than two in the metastatic setting. Abemaciclib was administrated at the dose of 200 mg every 12 h on a continuous schedule. The ORR (primary endpoint) was 19.7% (95% CI, 13.3–27.5), the CBR was 42.4%, mPFS was 6 months (95% CI 4.2–7.5) and median overall survival (OS) was 17.7 months (95% CI, 16 to not reached). In this study the most common ADRs were diarrhea, fatigue, nausea, neutropenia, leukopenia, anemia and increased serum creatinine (86). This trial led to the FDA approval of abemaciclib as single therapy in pretreated patients with HR+ HER2– MBC.

Finally, preliminary results from a Simon 2-stage single arm phase II trial in patients affected by HR+ HER2– MBC, NSCLC or melanoma with brain metastases showed a number of brain partial responses that met the predefined threshold for expanding the trial to stage 2. For each patient cerebrospinal fluid concentration of unbound abemaciclib were comparable and consistent with those in the plasma and tumor tissue (87). This trial provided evidence that abemaciclib is able to cross the BBB in human, coherently with preclinical evidence on mice xenografts (53). The second stage is ongoing.

#### Abemaciclib: Ongoing Trials

Several ongoing trials with single agent abemaciclib have completed patients' recruitment. An asian phase I study (NCT02014129) is evaluating the safety and toxicities of abemaciclib in advanced solid tumors and lymphomas in Japanese participants (88). Abemaciclib is also currently investigated in GBM at first relapse in the NCT02981940 phase II trial. Tumors must be pRb wild type and carry inactivation of CDKN2A/B or C in the tumor by homozygous deletion. The coprimary endpoint are the intratumoral abemaciclib concentration and the 6-months PFS (89). Another phase II trial (active but no more recruiting), the NCT02450539, is evaluating the efficacy of abemaciclib compared to docetaxel in patients with metastatic squamous NSCLC previously treated with platinum-based chemotherapy. The primary endpoint is PFS (90). A phase II ongoing study (NCT02308020), currently recruiting participants, is evaluating the activity and efficacy of abemaciclib in patients with brain metastases secondary to HR+ breast cancer, NSCLC or melanoma. The primary endpoint is the objective intracranial response rate. Preliminary results have been reported in a previous section of this review (87). Other ongoing trials are currently enrolling participants. More specifically, the NCT02919696 phase I trial is studying abemaciclib in native chinese patients with advanced and/or metastatic cancers (91). A phase II trial (NCT03130439) is also investigating the efficacy and activity of abemaciclib in metastatic triple negative breast cancer expressing pRb. The primary endpoint is the ORR (92). The NCT02846987 phase II trial is currently recruiting patients affected by not surgically resectable locally advanced or recurrent dedifferentiated liposarcoma with any number of prior therapies (including none). The primary endpoint is PFS (93). A biomarker-driven phase II study (NCT03356587) of abemaciclib in patients with recurrent or metastatic head and neck squamous cell carcinoma who failed to platinum-based therapy is also currently recruiting participants. Primary endpoint is response rate (94). Another phase II trial in (NCT03356223) patients with locally advanced/metastatic head and neck cancer is currently evaluating abemaciclib monotherapy after failure of platinum and cetuximab or anti-EGFR-based therapy, but only in tumors harboring a homozygous deletion of CDKN2A, and/or amplification of CCND1 and/or of CDK6. The primary endpoint is the 8-weeks non-progression rate (95). Finally, the NCT03310879 phase II study is testing abemaciclib in patients with solid tumors of non-breast origin harboring genetic alterations in genes encoding D-type Cyclins or amplification of CDK4/6 without therapeutic alternative. The progression-free rate at 4 months is the primary endpoint (96).

Ongoing trials for palbociclib, ribociclib, and abemaciclib are resumed in Table 3.

**Table 3:** Currently ongoing trials on CDK 4/6 inhibitors as single agent in solid tumors.

	**CDK4/6 inhibitor**	***N***	**Phase and setting**	**Primary endpoint(s)**
NCT03123744	Palbociclib	200	Non-randomized Phase II study of palbociclib in adult subjects with recurrent or refractory advanced cancers with aberration(s) in cyclin (CCN/CDK) signaling.	Response rates in subjects with advanced cancer and aberrations of cyclin pathway gene(s) who are treated with palbociclib
NCT02530320	Palbociclib	40	Phase II pilot, prospective, open label, multicenter clinical trial, to evaluate the safety and efficacy of palbociclib, in patients with oligodendroglioma or recurrent oligoastrocytoma anaplastic with the activity of the protein rb preserved	PFS, PFS6m
NCT03454919	Palbociclib	60	Phase II clinical study on efficacy of palbociclib in advanced acral melanoma with cell cycle gene aberrations	ORR, Complete response and partial response
NCT 03242382	Palbociclib	38	Phase II multicenter trial of palbociclib in second line of advanced sarcomas with CDK4 overexpression.	PFS rate
NCT03219554	Palbociclib	33	Phase II single center, open-label, single arm study of palbociclib treatment in patients with recurrent or metastatic advanced thymic epithelial tumor (TET) after failure of one or more cytotoxic chemotherapy regimens	PFS
NCT01907607	Palbociclib	63	Multicentre single-arm phase II study evaluating the efficacy and safety of orally Palbociclib, 125 mg/day, 21 days on/7 days off, in patients with documented disease progression while on therapy with second line sunitinib for unresectable and/or metastatic GIST.	Efficacy, assessed based on 4-months non-progression
NCT01356628	Palbociclib	23	Phase II study of Palbociclib in the treatment of patients with advanced hepatocellular carcinoma (HCC), a type of adenocarcinoma and the most common type of liver tumor.	Time to disease progression
NCT02806648	Palbociclib	21	Phase II trial to assess the activity and safety of Palbociclib in patients with well and moderately differentiated metastatic pancreatic neuroendocrine tumors (pNET)	Response rates
NCT01037790	Palbociclib	205	Phase II trial is studying the side effects and how well PD 0332991 works in treating patients with refractory solid tumors.	Response rates
NCT02345824	Ribociclib	3	Early-phase study to assess tumor pharmacokinetics and efficacy of the cdk4/6 inhibitor Ribociclib in patients with recurrent glioblastoma or anaplastic glioma	Inhibition of CDK4/CDK6 signaling pathway in cell proliferation
NCT03096912	Ribociclib	30	Phase II single arm study assessing efficacy and safety of Ribociclib in patients with advanced well-differentiated or dedifferentiated liposarcoma	Response to therapy as evaluated by RECIST 1.1 Response to therapy as evaluated by Choi [Time Frame: 36 months]
NCT02571829	Ribociclib	30	Phase II single arm study assessing efficacy and safety of Ribociclib in patients with advanced well-differentiated or dedifferentiated liposarcoma	Response to therapy as evaluated by RECIST 1.1 and Choi [Time Frame: 36 months (24 months accrual period and 12 months follow up period)]
NCT02300987	Ribociclib	10	Randomized, blinded, placebo-controlled, phase II trial of LEE011 in patients with relapsed, refractory, incurable teratoma with recent progression.	Progression free survival (PFS) [Time Frame: at 4 months]
NCT02919696	Abemaciclib	20	Phase I study of Abemaciclib in native Chinese patients with advanced and/or metastatic cancers.	Number of Participants with One or More Drug Related Adverse Events Number of participants with one or more drug related adverse events
NCT02014129	Abemaciclib	12	Phase I study of Abemaciclib in Japanese patients with advanced cancer	Number of Participants with LY2835219 Dose-Limiting Toxicities (DLT)
NCT02981940	Abemaciclib	36	Phase II study of Abemaciclib in recurrent glioblastoma	Intratumoral abemaciclib concentration [Time Frame: 2 years] PFS6m
NCT03130439	Abemaciclib	37	Phase II study of Abemaciclib for patients with retinoblastoma-positive, triple negative metastatic breast cancer.	Objective Response Rate [Time Frame: 2 years]ORR as confirmed Complete Response (CR) or Partial Response (PR) per Response Evaluation Criteria in Solid Tumors (RECIST)
NCT02846987	Abemaciclib	30	Phase II study of Abemaciclib in dedifferentiated liposarcoma	PFS [Time Frame: 12 weeks]
NCT03356587	Abemaciclib	32	Biomarker-driven, open label, single arm, multicentre phase II study of Abemaciclib in patients with recurrent or metastatic head and neck squamous cell carcinoma who failed to platinum-based therapy	Response rate [Time Frame: 24 months]
NCT03356223	Abemaciclib	25	Phase II trial aiming to evaluate the clinical interest of Abemaciclib monotherapy in patients with locally advanced/metastatic head and neck cancer after failure of platinum and Cetuximab or anti-EGFR-based therapy and harboring an homozygous deletion of cdkn2a, and/or an amplification of CCND1 and/or of CDK6	The 8-weeks non-progression rate defined as the rate of patients with complete response (CR), partial response (PR) or stable disease (SD) lasting at least 8 weeks, according to RECIST v1.1 [Time Frame: 8 weeks after start of treatment]
NCT02450539	Abemaciclib	150	Randomized phase II study of Abemaciclib vs. docetaxel in patients with stage iv squamous non-small cell lung cancer previously treated with platinum-based chemotherapy.	PFS
NCT02308020	Abemaciclib	247	Phase 2 study of Abemaciclib in patients with brain metastases secondary to hormone receptor positive breast cancer, non-small cell lung cancer, or melanoma.	Percentage of Participants Achieving Complete Response (CR) or Partial Response (PR): Objective Intracranial Response Rate (OIRR)
NCT03310879	Abemaciclib	38	Phase II study of the cdk4/6 inhibitor Abemaciclib in patients with solid tumors harboring genetic alterations in genes encoding D-type cyclins or amplification of CDK4 or 6.	Progression-free rate

## Conclusions

Albeit it is unquestionable, at present, that CDK 4/6i treatment proved to be more efficacious in combination strategies (e.g., in HR+ HER2– MBC is in combination with endocrine agents), the MONARCH 1 trial results (86) led to the FDA approval of abemaciclib as monotherapy for the treatment of adult patients with HR+ HER2– MBC with disease progression after prior ET and CT received in metastatic setting. This study opened up a new scenario for CDK4/6i, making them suitable as single agent treatment in heavily pretreated MBC. In this perspective, the TREND trial provided some evidence for some activity of palbociclib as single agent in pretreated patients with HR+HER2– MBC (65). A cross-trial comparison of response rate from the MONARCH-1 and TREND trial suggests that abemaciclib might be more effective than palbociclib in the same disease setting. However, this hint should be taken as hypothesis only, given the lack of direct comparisons between the two CDK4/6i. Additionally, there is a strong need for biomarkers predictive of response and resistance to better define which patients could benefit most from these drugs. In fact, mechanisms of resistance to CDK4/6i therapy have yet to be clearly identified. Laboratory evidences suggest that markers of intrinsic resistance might be the pRb loss and subsequent increase in p16^INK4A^, deregulation of cyclin E expression, E2F family members amplification and TP53 mutations (97). Interestingly, a study recently published from Condorelli et al. showed for the first time in human patients that acquired mutations leading to functional loss of pRb encoding gene (RB1) might emerge under treatment with palbociclib and ribociclib, maybe due to selective pressure from the CDK4/6i and might potentially confer therapeutic resistance (98). Results from ongoing trials in solid tumors will surely shed a light on CDK4/6i future development as single agents. It is likely that eventual new treatment indications might be acquired by the three inhibitors in the next future, especially in tumors where few therapeutic options are available, such as sarcomas.

## Author Contributions

All authors conceived the review. FS, ID, CR, and PD performed the literature search. FS drew the figures. FS, ID, and LD wrote the first draft of the manuscript. All authors revised and approved the final version of the manuscript.

### Conflict of Interest Statement

The authors declare that the research was conducted in the absence of any commercial or financial relationships that could be construed as a potential conflict of interest.

## Abbreviations

CDK4/6: cyclin-dependent kynases 4 and 6
CDK4/6i: CDK4/6 inhibitors
CCND: cyclin D
PI3K: phosphatidylinositol 3-kinase
mTOR: mammalian target of rapamycin
MAPK: mitogen-activated protein kinase
JAK: janus kinase
STAT: signal transducer and activator of transcription
NF-kB: nuclear factor kappa-light-chain-enhancer of activated B cells
miRNAs: microRNAs
pRb: retinoblastoma protein
ET: endocrine therapy
ER+: estrogen receptor positive
HR+: hormone receptor positive
HER2–: human epidermal growth factor receptor 2 negative
MBC: metastatic breast cancer
BC: breast cancer
GBM: glioblastoma multiforme
WD/DDLS: well-differentiated/dedifferentiated liposarcoma
NSCLC: non-small cell lung cancer
SCLC: small cell lung cancer
GIST: gastrointestinal stromal tumors
PDA: pancreatic ductal adenocarcinoma
EAC: esophageal adenocarcinoma
BBB: blood-brain barrier
ORR: overall response rate
PFS: progression-free survival
mPFS: median progression-free survival
TTP: time to progression
DCR: disease control rate
CBR: clinical benefit rate
DLT: dose-limiting toxicity
MTD: maximum tolerated dose
RP2D: recommended phase II dose
ADRs: adverse reactions
SD: stable disease
PR: partial response
CR: complete response
CI: confidence interval
HR: hazard ratio.
